# Alkali-Activated Materials from Diverse Solid Precursors: Structural, Mechanical and Radiological Properties

**DOI:** 10.3390/gels12030200

**Published:** 2026-02-27

**Authors:** Nataša Mladenović Nikolić, Marija Ivanović, Snežana Nenadović, Jelena Potočnik, Sabina Dolenec, Dušan Bučevac, Aleksandar Kandić, Ljiljana Kljajević

**Affiliations:** 1 Department of Materials, “Vinča” Institute of Nuclear Sciences—National Institute of the Republic of Serbia, University of Belgrade, 11000 Belgrade, Serbia; marija@vin.bg.ac.rs (M.I.); msneza@vin.bg.ac.rs (S.N.); bucevac@vin.bg.ac.rs (D.B.); ljiljana@vin.bg.ac.rs (L.K.); 2 Department of Atomic Physics, “Vinča” Institute of Nuclear Sciences—National Institute of the Republic of Serbia, University of Belgrade, 11000 Belgrade, Serbia; jpotocnik@vin.bg.ac.rs; 3 Slovenian National Building and Civil Engineering Institute, Dimičeva Ulica 12, 1000 Ljubljana, Slovenia; sabina.dolenec@zag.si; 4 Department of Geology, Faculty of Natural Sciences and Engineering, University of Ljubljana, Aškerčeva Ulica 12, 1000 Ljubljana, Slovenia; 5 Department of Nuclear and Plasma Physics, “Vinča” Institute of Nuclear Sciences—National Institute of the Republic of Serbia, University of Belgrade, 11000 Belgrade, Serbia; akandic@vin.bg.ac.rs

**Keywords:** wood ash, metakaolin, alkali-activated materials, radionuclides

## Abstract

This study investigates the gel characteristics of alkali-activated materials (AAMs) synthesized using wood ash (WA), and metakaolin (MK) as solid precursors. The research explores the influence of precursor type and sodium hydroxide (NaOH) concentrations in the alkali activator solution on the resulting physicochemical, microstructural, mechanical, and radiological properties of gels. The alkaline activators were prepared by mixing sodium hydroxide solutions (6 M and 12 M) with a sodium silicate (water glass) solution at a volume ratio of 1.5. The physicochemical characteristics of raw materials and AAMs were thoroughly analyzed using X-ray fluorescence (XRF), Diffuse Reflectance Infrared Fourier Transform (DRIFT) spectroscopy, X-ray diffraction (XRD), and scanning electron microscopy (SEM) with EDS elemental mapping. FTIR analysis confirmed the formation of an amorphous gels geopolymer network. XRD revealed the presence of characteristic crystalline phases (quartz, calcite) within an amorphous matrix. Mechanical properties, such as compressive strength, depended on precursor type and alkali molarity: metakaolin (12 M) reached ~14 MPa, while wood ash showed ~4 MPa (6 M) and ~0.5 MPa (12 M) due to high CaO, low Si and Al, and unfavorable SiO_2_/Al_2_O_3_ (5.71) and Na_2_O/Al_2_O_3_ (3.19) ratios. Furthermore, this research estimates radiological doses by quantifying radionuclide content via gamma-spectrometry. Alkali activation significantly reduced radiological hazard parameters, with radium equivalent activity (*Ra_eq_*) decreasing to 238.0 Bq/kg and the external hazard index (*H_ex_*) to 0.643 for A_12_MK, while the annual effective dose rate for A_12_WA was only 0.265 nSv/y-all values remaining well below the recommended safety limit of 370 Bq/kg (≤1 mSv/y). The decrease in activity concentration index (*I_γ_*), *Ra_eq_*, and *H_ex_* with increasing NaOH concentration indicates effective radionuclide immobilization within the geopolymer matrix, confirming the suitability of these alkali-activated materials for safe use in construction from a radiation protection perspective.

## 1. Introduction

Alkali-activated materials (AAMs) represent an essential innovation for a more sustainable future in construction. Their ability to minimize carbon footprint, valorize industrial waste, and provide long-lasting, resilient building solutions makes them a powerful tool in the global effort to combat climate change and promote a greener economy [1,2,3]. AAMs have emerged as promising sustainable alternatives to ordinary Portland cement (OPC) due to their potential to reduce greenhouse gas emissions and valorize industrial and agricultural by-products. With the cement sector responsible for a large proportion of global CO_2_ emissions, the innovation of low-carbon binders like AAMs plays a pivotal role in the development of eco-friendly construction materials. The most commonly used industrial by-products in cement and concrete manufacturing include coal fly ash (CFA), ground granulated blast furnace slag (GGBS), silica fume (SF), and calcinated clays like metakaolin (MK) [4,5,6,7]. Precursors such as fly ash (FA) and metakaolin (MK) are rich in aluminosilicate phases essential for geopolymerization, offering diverse sources for alkali activation.

Wood ash is a by-product of biomass, while metakaolin, produced by calcining kaolin clay, is well-regarded for its high reactivity and pozzolanic properties [8,9]. According to a published report by representatives from several European countries and Canada [10], grate combustion is predominantly used for the combustion of biomass. According to the European Waste Catalogue and hazardous residues list [11], both bottom ash and fly ash coming from the combustion of untreated wood are classified as non-hazardous wastes. Woody biomass bottom ash may be reused as a building material for replacing granular material in geotechnical works, like road foundations [12]. Its application to agricultural or forest soils has also been proposed [13,14]. Metakaolin (MK) is an amorphous aluminosilicate fine, white powder obtained as a result of kaolinite clay calcination at high temperatures from 450 °C to 900 °C, depending on the purity and crystallinity of the precursor clay. MK is mostly used in several architectural applications and high-performance concrete [15,16,17]. The structure and properties of the resultant materials depend critically on factors including precursor chemistry, activator composition, concentration, and curing conditions, as well as various combinations of mixing and processing parameters. These parameters govern the degree of dissolution, gel formation, microstructural development, and ultimately physical properties, which have a critical impact on AAMs [8,9,18]. In addition to mechanical performance, multifunctional properties such as radiological safety and environmental stability are increasingly recognized as vital for broadening AAMs’ applications beyond conventional construction.

Alkali-activated materials based on solid precursors, such as wood ash and metakaolin, are produced through chemical reaction with alkalis. Commonly used alkalis are sodium hydroxide in combination with sodium silicates. These reactions generate gels with an amorphous Si-O-Al polymer structure [19]. Based on the calcium content, the AAMs are classified as high-calcium system (form an alkali calcium-aluminosilicate hydrate (C− (N) −A−S−H) gel), low-calcium system (form a three-dimensional hydrous alkali-aluminosilicate (N-A-S-H) gel, and medium-calcium system (characterized by the coexistence of both C− (N)−A−S−H and N−A−S−H gels). Beyond these main products, AAMs generate various secondary products influenced by the precursor’s composition, the specific alkaline activator used, and the applied curing conditions [20].

A study conducted by S.S. Narani et al. (2024) [21] investigates the development of alkali-activated materials based on wood fly ash and ground granulated blast furnace slag. The results indicate that wood fly ash, due to its low mechanical resistance, is not suitable for standalone use in construction. However, it demonstrates exceptional potential for waste stabilization, while achieving structural properties requires its combination with other precursors, such as blast furnace slag [21]. The experimental work of M. Kaya et al. (2026) [22] explored the influence of key parameters-specifically Na dosage (10, 14, and 18%), ceramic powder content (0, 10, 20, and 30%), and curing temperatures (20, 70, and 100 °C)-on geopolymerization kinetics. Their analysis revealed that 100 °C curing optimizes the formation of a compact geopolymeric matrix, although strength loss was observed at very high NaOH molarities or ceramic powder levels. These results emphasize the importance of balancing precursor ratios and activator concentration to avoid microstructural instability [22]. S. Jurado Contreras et al. (2022) [8] analyzed the physical, mechanical, and microstructural properties of metakaolin-based bricks across a broad range of biomass fly ash contents (25–100 wt.%). The researchers investigated the role of precursor alkali content—modified via washing—and activator molarity, establishing that optimal strength is achieved with a 50 wt.% biomass fly ash replacement and 8 M NaOH activation, primarily due to the supplementary alkalis released from the ash [8].

Efficient utilization of waste biomass ash helps safeguard the environment, conserve natural resources, and minimize landfill reliance. Especially, the combustion of wood generates waste materials, primarily ash, that can severely threaten both the environment and public health through contamination and safety risks. To ensure health and safety, a radiological evaluation is essential before utilizing wood ash in building applications.

Radiological assessments ensure the safe use of waste-derived materials. Comprehensive characterization combining physicochemical, mechanical, and radiological analyses is therefore essential to optimize design and expand the utility of AAMs. Nevertheless, the widespread application of AAMs is hindered by the complexity and time intensity of experimental designs. Despite advances, challenges remain in correlating precursor variations and alkali concentrations with multifunctional outcomes, especially as complex interactions influence the material’s gel structure and crystallinity.

In this study, materials from WA and MK were synthesized under varying NaOH concentrations, employing techniques such as XRF, DRIFT, XRD, and SEM for in-depth microstructural insights. Furthermore, the study integrates mechanical testing and radiological dose calculations to elucidate gel structure-property-function relationships. By providing a multifunctional perspective, this research contributes to the rational development of alkali-activated materials with tailored properties for sustainable construction and environmental applications.

## 2. Results and Discussion

### 2.1. Characterization of Precursors and Alkali-Activated Materials

To enable a comprehensive understanding of the alkali activation process, the chemical composition of the precursors and the resulting alkali-activated materials, along with the particle size distribution of the precursors, were examined.

#### 2.1.1. Chemical Composition of Precursors and Alkali-Activated Materials

According to our previous work [23], the chemical composition of precursors WA and MK shows that the dominant compound in the WA precursor is CaO, while the MK precursor is mainly composed of Al_2_O_3_ and SiO_2_ (Table 1). The chemical composition of selected AAMs (A_6_WA, A_6_MK and A_6_MKWA) is presented in Table 2.

After the alkali activation process, it can be observed (Table 2) that A_6_WA still have the high value of CaO, while A_6_MK has a high value of Al_2_O_3_ and SiO_2_. The SiO_2_/Al_2_O_3_ and Na_2_O/Al_2_O_3_ ratios in A_6_WA are 5.71 and 3.19, respectively. The SiO_2_/Al_2_O_3_ ratios is 2.30, whereas Na_2_O/Al_2_O_3_ ratio is 0.36 for A_6_MK. Incorporating 10 wt.% WA into the A_6_MK system (sample A_6_MKWA) notably increased the CaO levels. The resulting chemical landscape is characterized by ratios of 2.30 and 0.34, indicating a specific geopolymeric stoichiometric balance.

#### 2.1.2. Particle Size Distribution of Precursor and Alkali-Activated Materials

Particle size distribution was evaluated for precursors and the selected alkali-activated materials (AAMs) using laser diffraction analysis. According to the analysis, particle size parameters of precursors (Table 3) and AAMs (Table 4) are characteristic percentiles *d*_0.1_, *d*_0.5_ and *d*_0.9_. These values correspond to the particle diameters below which 10%, 50% and 90% of the cumulative volume distribution fall, respectively. Before obtaining these measurements, each sample was subjected to dry dispersion to minimize agglomeration.

Particle size distribution measurements of metakaolin indicate that 10% of the particles (d_0.1_) have diameters smaller than 4.054 µm, 50% of the particles (d_0.5_) have diameters smaller than 122.118 µm, and 90% of the particles (d_0.9_) have diameters smaller than 383.889 µm. Compared to metakaolin, wood ash consists of finer particles, with 10%, 50%, and 90% of the particles (d_0.1_, d_0.5_, and d_0.9_) having diameters smaller than 0.955 µm, 5.073 µm, and 70.781 µm, respectively.

For the A_6_WA sample, the particle size distribution exhibited d_0.1_= 14.517 µm, d_0.5_ = 120.636 µm, and d_0.9_ = 321.955 µm. In the case of A_6_MK, 10% of the particles were smaller than 21.395 µm, the median particle diameter was 178.884 µm, and 90% of the particles were below 382.181 µm. The A_6_MKWA sample showed a finer fraction at the lower percentile with d_0.1_ = 7.939 µm, while the median and coarse fractions were d_0.5_ = 122.793 µm and d_0.9_ = 331.630 µm. These results indicate notable differences in the fine and coarse fractions among the investigated AAM precursors, which may influence reactivity, packing density, and overall performance in alkali-activation systems.

#### 2.1.3. Structural and Phase Characterization of Precursors

**Figure 1:**
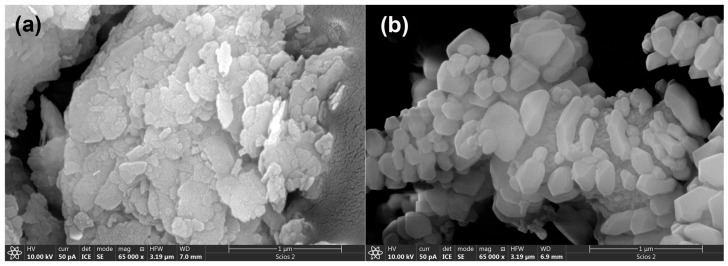
SEM micrographs of precursors: (**a**) MK and (**b**) WA.

The structural characterization (XRD and FTIR analyses) of the precursors (MK and WA) has been previously reported in our research paper [23]. WA was composed mainly of calcite (PDF No. 01-071-3699), larnite (PDF No. 01-083-0464), and portlandite (PDF No. 00-044-1481). Portlandite typically forms at low to moderate combustion temperatures as a result of calcite decomposition [23]. For MK, in addition to the characteristic amorphous hump observed in the 2*θ* range of 20–40°, muscovite (PDF No. 01-070-975), illite (PDF No. 00-043-0685), dominant quartz (PDF No. 00-033-1161), and residual crystalline kaolinite (PDF No. 01-072-5860) were identified [23]. Figure 1 presents SEM micrographs of WA and MK samples.

As illustrated in Figure 1a, WA is composed of a network of intertwined chains of irregular yet uniformly sized particles, generally smaller than 1 μm. The smallest particles aggregate to form larger structures, with edges ranging from angular to rounded, while the interiors of the larger particles are filled with finer ash fragments. In comparison (Figure 1b), MK exhibits a dense, compact matrix of particles with varied morphologies, predominantly flake-like in appearance.

### 2.2. Structural and Phase Characterization of Alkali-Activated Materials

DRIFT and XRD analyses were performed on the alkali-activated materials to investigate their structural features and phase composition. These analyses provide insight into the type and extent of gel formation resulting from alkali activation.

**Figure 2:**
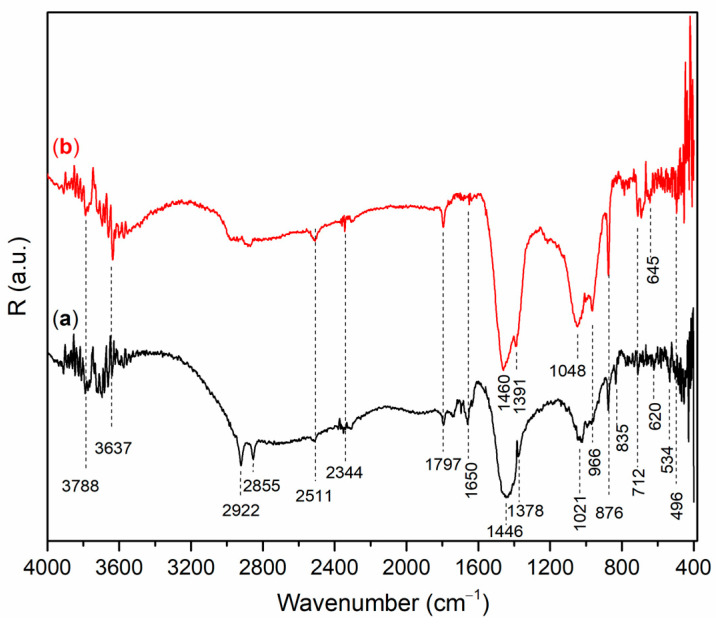
DRIFT spectrum of A_6,12_WA: (**a**) A_6_WA and (**b**) A_12_WA.

**Figure 3:**
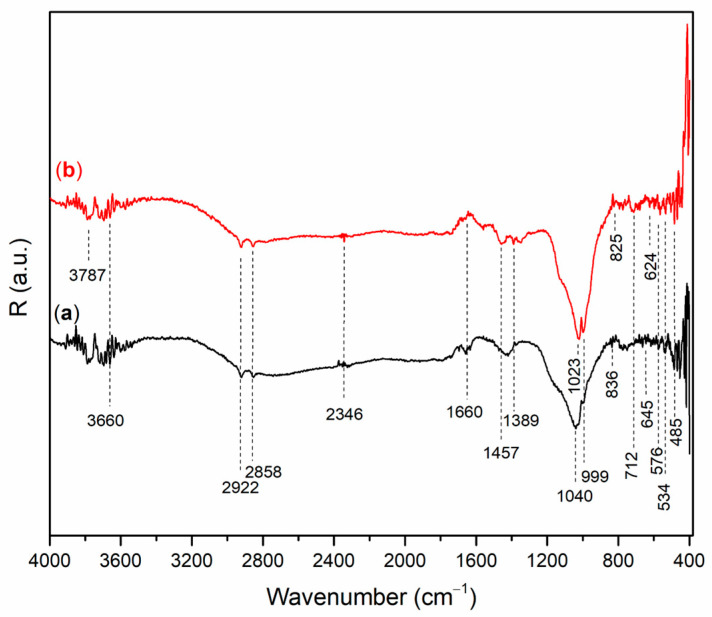
DRIFT spectrum of A_6,12_MK: (**a**) A_6_MK, and (**b**) A_12_MK.

Figure 2 and Figure 3 show the DRIFT spectra of AAMs based on wood ash (A_6,12_WA), metakaolin (A_6,12_MK).

All DRIFT spectra show peaks in the range from 3400 cm^−1^ to 3600 cm^−1^, which belong to the vibrations of OH groups in the material structure, as well as a peak in the range from 1660 cm^−1^ to 1640 cm^−1^, which indicates adsorbed and/or bound water molecules [24,25]. Also, the spectrum of AAMs possesses a peak in the range from 1100 cm^−1^ to 800 cm^−1^, and these peaks are assigned to stretching vibrations of Si−O bonds [26].

The bands at 2922 cm^−1^ and 2855 cm^−1^ observed in A_6_WA (Figure 2b) are associated with the asymmetric and symmetric stretching of methyl and methylene groups, while the weak bands at 2344 cm^−1^, present in all alkali-activated wood ash samples, are attributed to the presence of HCO_3_^−^ ions [23,27,28]. The bands appearing in the range of 1480 cm^−1^ to 1440 cm^−1^ are characteristic of C–O stretching vibrations [29], while the peaks at 2511 cm^−1^ and 1797 cm^−1^ are associated with CO_3_^2−^ vibrations and are attributed to the presence of carbonates [30].

Symmetric stretching vibrations of Si–O–Si/Si–O–Al were observed at 1048 cm^−1^ in A_12_WA (Figure 2b), respectively, while in A_6_WA (Figure 2b) this stretching appeared at a lower wavenumber of 1021 cm^−1^ [31,32]. The peak at 966 cm^−1^ corresponds to asymmetric stretching vibrations of Si–O–Si and Si–O–Al [33,34]. A sharp peak at 876 cm^−1^ indicates the symmetric stretching of AlO_4_^−^ groups in Al–O–Si bonds within the alkali-activated material [35], while the peak at 712 cm^−1^ indicates the presence of calcite [36]. Vibrational modes around 500 cm^−1^ are indicative of Si–O–Si bending [37]. In alkali-activated materials based on calcium-rich wood ash, Ca participates in the formation of Ca–O–Si and Ca–O–Al bonds, particularly during geopolymerization, leading to the development of C–S–H and C–A–S–H phases. These bonds are typically observed as broad bands in the regions of 900–850 cm^−1^ (Si–O–Ca stretching) and 500–460 cm^−1^ (Ca–O vibrations), often overlapping with Si–O–Si and Si–O–Al vibrations [29,38,39].

The symmetric stretching vibrations of Si–O–Si/Si−O−Al (Figure 3) is observed in the region between 1040 cm^−1^ and 990 cm^−1^ [40]. According to Figure 3a, A_6_MK, a band is observed at 1040 cm^−1^, which shifts toward a lower wavenumber (1023 cm^−1^) in A_12_MK (Figure 3b). The band at 1457 cm^−1^, observed in alkali-activated metakaolin samples, corresponds to O–C–O bonds in carbonate groups formed as a result of the reaction between alkali metal hydroxides and atmospheric CO_2_. Stretching vibrations of Si–O–Si and Si–O–Al bonds are observed at 999 cm^−1^. In the alkali-activated A_6_MK sample, a band appears at 836 cm^−1^, which shifts toward a lower wavenumber (825 cm^−1^) in A_12_MK, and is associated with stretching vibrations of tetrahedrally coordinated Al–O [41]. The highly cross-linked structure of the alkali-activated materials, formed after the reaction between MK and the alkali activation solution, gives rise to a band at 712 cm^−1^ corresponding to the formation of Si–O–Al(IV) bonds [40]. The band at 576 cm^−1^ is associated with the symmetric stretching and bending vibrations of T–O–T bonds (T = Si or Al). The band in the region around 534 cm^−1^ corresponds to Si–O–Al(VI) bonds formed during the alkali activation process [42]. Vibrations at 485 cm^−1^ can be attributed to T–O bending modes, where T represents Si or Al [43].

**Figure 4:**
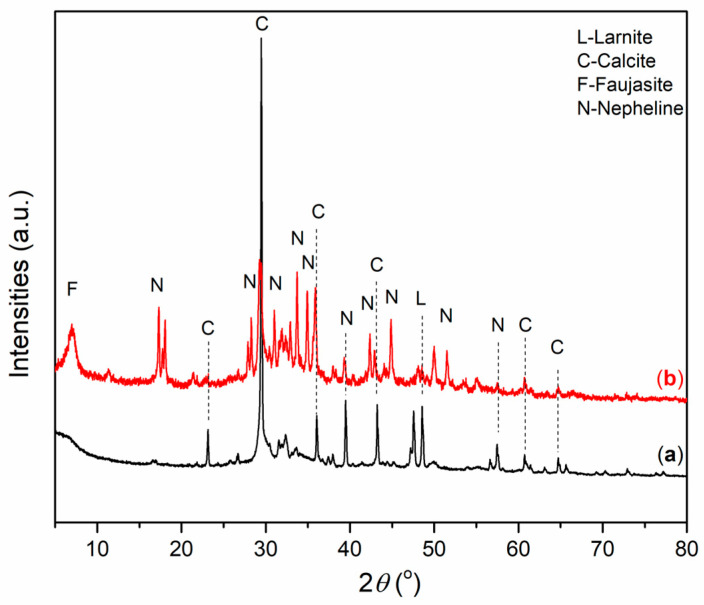
XRD diffractogram of A_6,12_WA: (**a**) A_6_WA, and (**b**) A_12_WA.

**Figure 5:**
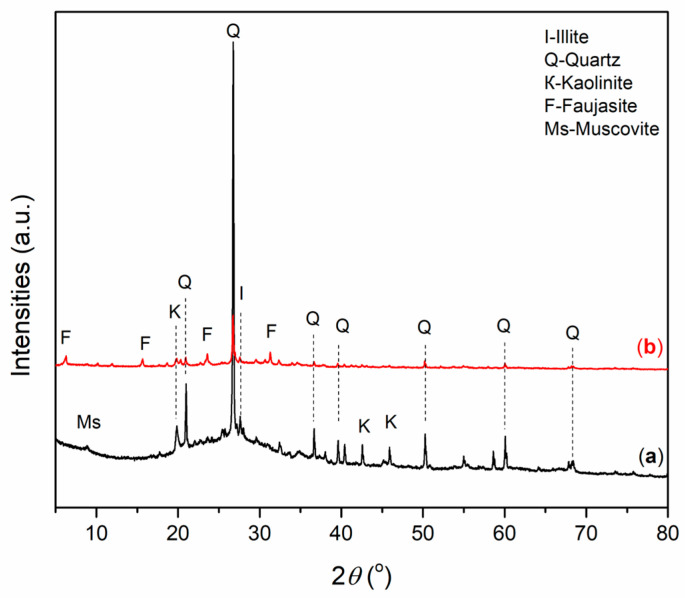
XRD diffractogram of A_6,12_MK: (**a**) A_6_MK, and (**b**) A_12_MK.

Mineralogical analysis of alkali-activated materials was performed in order to identify the formation of mineral phases of new alkali-activated materials. The X-ray diffraction patterns of AAMs based on wood ash (A_6,12_WA) and metakaolin (A_6,12_MK) are presented in Figure 4 and Figure 5.

XRD analysis of alkali-activated wood ash (Figure 4) shows that the A_12_WA samples (Figure 4b) exhibit the most significant amorphous hump between the 2*θ* range of 25–40°. Alkali activation of WA leads to dissolution of portlandite and larnite [23], releasing Ca^2+^, Si and Al and forming amorphous gels. Also, the crystalline phases of calcite, larnite and nepheline are formed, with peak intensity increasing with NaOH molarity, while in the A_12_WA, faujasite appears due to the extremely high pH. Higher NaOH molarity accelerates reactions, enhances crystallization, and promotes the formation of additional phases, while Na_2_SiO_3_ stabilizes gels and improves the microstructure. Furthermore, nepheline was identified through narrow, well-defined peaks, suggesting a highly ordered structural arrangement of the crystalline minerals. This is particularly evident in the A_12_WA sample (Figure 4b), where a distinct nepheline peak is observed [44,45]. Although nepheline was identified based on database reference [46], it cannot be reliably concluded that it forms in AAMs solely through alkali activation and curing at ~60 °C, without additional heating or sintering. Further research is needed in this regard. Given the low Si and Al contents, the extremely high NaOH molarity, and a 28-day aging period, favorable experimental conditions for its formation may have developed, and this is one possible explanation.

An amorphous phase was observed in A_6_MK (Figure 5a), characterized by an elevated background in the diffractogram within the range of 18–32° 2*θ*, along with diffraction peaks corresponding to kaolinite and quartz as the dominant crystalline phases. In A_6_MK (Figure 5a), a muscovite reflection was observed, while in A_12_MK (Figure 5b), a phase was identified that most likely formed as a result of the increased NaOH concentration, which enhances the activity of OH^−^ ions, and corresponds to a zeolite-type phase, namely faujasite [42,47]. An illite peak was also identified, representing an associated mineral from the clay mineral group [48].

Structural, DRIFT, and mineralogical analyses of the alkali-activated metakaolin–wood ash system (MKWA) have been reported previously [23]. The crystalline phases largely corresponded to those present in metakaolin, while muscovite was absent in AWAMK. The contribution of crystalline phases from wood ash was not observed, likely due to its low proportion in the mixture. Variation in the molarity of the activator did not induce the formation of new crystalline phases. A weak band at 849–878 cm^−1^, observed in that alkali activated samples, was assigned in the DRIFT spectra of MKWA to the bending vibrations of Si–OH groups. The presence of Si–OH indicates incomplete polycondensation, which correlates with reduced compressive strength in A_6_ and A_12_WAMK materials [23,49].

In alkali-activated materials, crystalline phases identified by XRD analysis, such as larnite and calcite, i.e., can notably influence the characteristics of the gel phase. Ca-, Na-, or Al-rich crystalline compounds release ions that can integrate into the developing gel networks, modify stoichiometry, and increase matrix densification. Crystalline phases can also serve as nucleation sites, accelerating gel creation, although extreme or unevenly distributed crystals may disorder gel progression, increase porosity, and influence microcracking. For that reason, moderate crystallinity mainly intensifies gel homogeneity and compressive strength, and excessive crystallization can compromise matrix integrity and the extended durability of the material.

The XRD patterns provide key information on gel formation in the alkali-activated materials. The broad amorphous hump (≈18–40° 2*θ*), especially pronounced in A_12_WA (≈25–40° 2*θ*) and A_6_MK (≈18–32° 2*θ*), confirms the formation of geopolymeric gels responsible for strength development. In wood ash systems, dissolution of Ca-rich phases promotes the formation of C–S–H and C−A−S−H gels [50], while increased NaOH molarity enhances crystallization (calcite, nepheline, faujasite), indicating partial transformation of the amorphous gel and possible reduction in homogeneity.

In metakaolin-based materials, the amorphous halo confirms N–A–S–H gel formation [51], while the appearance of zeolitic phases (e.g., faujasite) at higher alkalinity reflects increased precursor dissolution and structural reorganization [52].

### 2.3. Mechanical Properties

The mechanical properties of alkali-activated materials derived from WA and MK precursors were evaluated to assess the influence of precursor composition, structural formation, and microstructural characteristics on compressive strength, with a representative MK–WA mixture containing 10 wt.% WA, activated with 6 M and 12 M NaOH, selected for further evaluation of compressive, microstructural, and radiological properties, while its structural features have been previously reported [23].

#### 2.3.1. Compressive Strength of Alkali-Activated Materials

**Figure 6:**
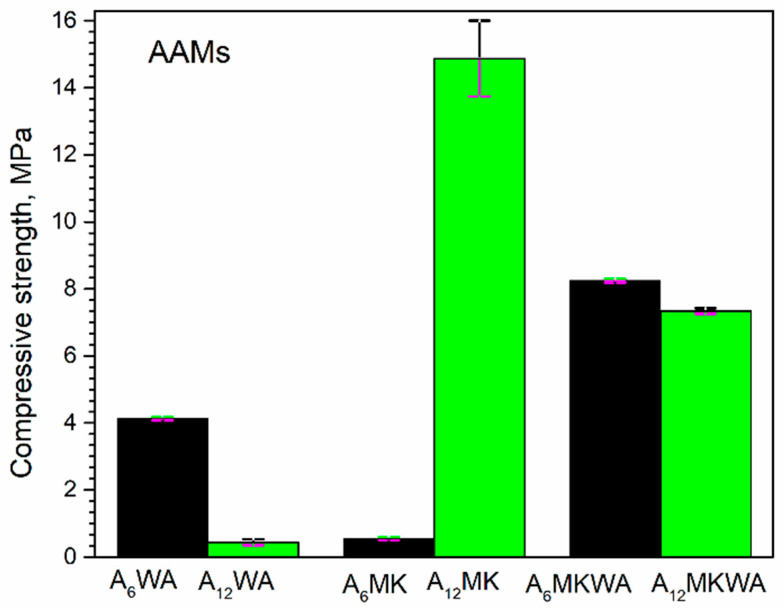
Compressive strength of alkali-activated materials, alkali-activated wood ash-A_6_WA and A_12_WA; alkali-activated metakaolin-A_6_MK and A_12_MK and two-component alkali-activated wood ash and metakaolin-A_6_MKWA and A_12_MKWA.

The compressive strength results of AAMs combined with post-activation chemical analysis show a strong dependence on precursor type, alkali concentration, and molar ratios. Figure 6 shows the compressive strength of alkali-activated materials, A_6,12_WA, A_6,12_MK and A_6,12_MKWA.

WA is characterized by high CaO content, while A_6_WA shows elevated SiO_2_/Al_2_O_3_ (5.71) and Na_2_O/Al_2_O_3_ (3.19) ratios. Such composition favors the formation of calcium phases, such as calcium silicate hydrates (C-S-H), which do not effectively contribute to the three-dimensional Si–O–Al network. This explains the poor performance of WA in the 12 M system, consistent with literature indicating that high-calcium precursors may reduce mechanical properties under strong alkali conditions [53,54,55]. WA has very high CaO content (~60%) and very low Si and Al contents (~4%), which prevents the formation of the characteristic three-dimensional N–A–S–H gel. The reaction primarily involves hydration and precipitation of calcium phases, forming Ca(OH)_2_ and partially C–S–H/C–A–S–H gels. Wood ash activated with 6 M NaOH/Na_2_SiO_3_, a small amount of binding gel is formed, giving a strength of ~4 MPa. Increasing NaOH to 12 M causes excessive Ca dissolution, destabilizes gels, and increases porosity, resulting in a drastic strength reduction (~0.5 MPa).

MK is significantly more reactive than WA. In metakaolin alkali-activated with 6 M NaOH/Na_2_SiO_3_ solution, the reaction proceeds quickly, but the resulting gels are partially heterogeneous (see Figure 8a) and less polymerized, leading to low strength (~0.5 MPa). In materials alkali-activated with 12 M NaOH/Na_2_SiO_3_ activation solution, better homogenization and dense gel formation occur (see Figure 8b), increasing strength to ~14 MPa (Figure 6). Metakaolin’s high reactivity is due to its amorphous Si and Al content. A_6_MK has relatively low SiO_2_/Al_2_O_3_ (2.30) and Na_2_O/Al_2_O_3_ (0.36) ratios, but high Al_2_O_3_ and SiO_2_ contents. Under strong alkali conditions, these characteristics allow the formation of a dense Si–O–Al network, explaining the significantly higher compressive strength in the 12 M system. These observations align with previous studies on metakaolin-based geopolymers [3,56,57,58,59,60,61]. Thermal curing (48 h at 60 °C) promotes geopolymerization in MK but accelerates water evaporation and Ca(OH)_2_ precipitation in WA, further increasing heterogeneity and microcracking.

Alkali activation with 6 M NaOH in alkali solution, with the addition of 10 wt.% of wood ash, shows a higher value of compressive strength compared to alkali-activated wood ash and metakaolin. Substituting a portion of metakaolin with 10 wt.% wood ash, activated by a 6 M NaOH solution, can enhance compressive strength compared to pure metakaolin or wood ash-based alkali-activated systems. Wood ash, characterized by its significant CaO and K_2_O content, functions as both a chemical reactant and a physical filler. At a 10 wt.% dosage, the ash particles effectively densify the matrix interfaces, promoting a more compact microstructure through the synergistic formation of N−A−S−H (sodium aluminosilicate hydrate) and C−(A)−S−H (calcium-modified) gel frameworks [62]. The calcium inherent in wood ash provides additional nucleation sites that accelerate the geopolymerization of the metakaolin-based materials [63].

Studies emphasize the critical role of optimal Si/Al and Na/Al ratios in alkali-activated materials. Metakaolin (MK), with Si/Al ratios close to recommended values, promotes efficient geopolymerization and formation of dense three-dimensional Si–O–Al networks. In contrast, wood ash (WA), with high Si/Al and Na/Al ratios and elevated CaO content, favors the formation of non-structural calcium phases, compromising mechanical properties [57,58]. The Si/Al molar ratio is particularly important, as these materials consist of amorphous to semi-crystalline silico-aluminate networks. Previous studies have shown that altering the Si/Al ratio in alkali-activated systems significantly affects the compressive strength of AAMs. Davidovits [64] proposed an optimal SiO_2_/Al_2_O_3_ ratio of 3.5–4.5. These findings indicate that improving geopolymer performance can be achieved by selecting appropriate precursors and alkali activators to maintain the Si/Al molar ratio within the optimal range [65].

Another important factor contributing to low strength is the high porosity resulting from a high liquid/solid (L/S) ratio and uneven curing. Rapid setting in a highly alkaline environment leads to heterogeneous microstructures and microcracking. As a consequence of these combined effects, the dense matrix remains insufficiently developed, with unreacted precursor particles and weak gel structures prevailing. Therefore, even at higher NaOH molarity (12 M), compressive strengths typical of fully developed geopolymers are not achieved. In short, the main reasons for the low compressive strength of these systems are incomplete precursor activation and high structural porosity, which hinder full geopolymerization.

The primary cause of low compressive strength in wood ash-based systems lies in their chemical composition—extremely high calcium content, combined with the lack of a sufficient amount of Al and Si species (due to the low Si and Al content in WA). In such a system, alkali activation does not lead to true geopolymerization but rather to simple hydration and calcium phase precipitation, which cannot form a compact binding matrix. Consequently, increasing NaOH molarity does not increase strength but probably destabilizes the reaction structure and reduces mechanical performance. These findings highlight that the mechanical properties of AAMs depend on the interplay of precursor chemistry, alkali activator concentration, and post-activation molar ratios. Amorphous and reactive precursors (MK) are suitable for high-strength geopolymers under strong alkali conditions, whereas calcium-rich or less reactive precursors (WA) require lower alkalinity, thermal or chemical modification, or blending with silicon-rich materials to improve compressive strength [66,67].

#### 2.3.2. Microstructural Features Related to Compressive Strength

Alkali-activated materials (AAMs), whether derived from pure MK or WA precursors or from MK–WA mixtures, generally form a dense, predominantly amorphous gel matrix composed mainly of N−A−S−H or C−A−S−H gel phases, depending on the calcium content of the precursor [68], in which unreacted or partially reacted precursor particles are embedded. The gel contains pores of varying sizes and connectivity, while small crystalline inclusions may be present within the amorphous network. These microstructural features play a critical role in determining compressive strength and are further examined here using SEM analysis to elucidate the influence of gel continuity, porosity, and particle distribution on mechanical performance.

**Figure 7:**
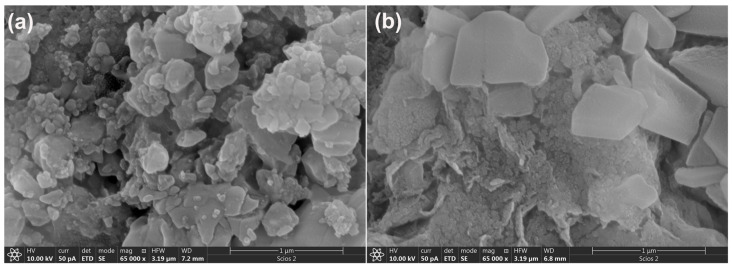
SEM micrographs of A_6,12_WA: (**a**) A_6_WA, and (**b**) A_12_WA.

The morphology of the alkali-activated wood ash samples (A_6,12_WA) was investigated using scanning electron microscopy, and the representative micrographs are presented in Figure 7. The SEM images reveal that the microstructure of the samples evolves progressively with increasing NaOH concentration in the alkali activator solution. The alkali-activated wood ash exhibits irregularly distributed agglomerates with uneven, rounded particle boundaries. The coexistence of distinctly porous regions alongside more compact domains indicates a pronounced heterogeneity across the sample surface, which is consistent with the observations reported by Brigolini Silva et al. (2021) [69]. The observed heterogeneity and crystal distribution can be attributed to the high calcium content of wood ash (approximately 39%) [17]. Calcium oxide accelerates the dissolution of reactive components and promotes the rapid formation of calcium–silicate and calcium–aluminate hydrate phases (C−S−H, C−A−H gels), yielding a structure more similar to Ca-rich alkali-activated materials than to classical geopolymer systems. XRD analysis (Figure 2) confirms that, in addition to the amorphous gel structure, the crystalline phases calcite, larnite, and nepheline are present in A_6,12_WA samples. Larnite commonly appears as needle-like granules, calcite as platy or polygonal crystals, and nepheline as finely dispersed crystals. In highly alkaline solutions of alkali activator (12 M NaOH), the crystalline phase of faujasite with a lamellar structure is also observed. The porosity and crystal size depend on the alkali activator molarity: lower molarity produces a loose gel with small, dispersed crystals; medium molarity results in a denser gel with larger crystals; and high molarity leads to a compact gel matrix with well-defined crystals.

**Figure 8:**
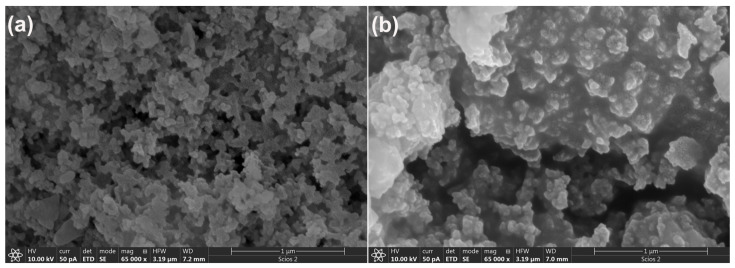
SEM micrographs of A_6,12_MK: (**a**) A_6_MK, and (**b**) A_12_MK.

Following the analysis of alkali-activated wood ash, the morphological features of alkali-activated metakaolin A_6,12_MK were examined to assess the effect of NaOH concentration. The obtained SEM micrographs are shown in Figure 8. The results reveal that varying the activator concentration leads to notable changes in surface morphology. In alkali-activated A_6_MK (Figure 8a), the surfaces are relatively irregular but maintain a dispersed arrangement of rounded particles. With increasing NaOH concentration, the A_12_MK sample (Figure 8b) exhibits a more uniform surface, where individual rounded particles coalesce into larger, block-like structures with well-defined boundaries. This progressive morphological evolution reflects the influence of higher alkalinity on the nucleation and growth of reaction products, and is consistent with previous observations reported by Yi Liu et al. (2016) [70].

**Figure 9:**
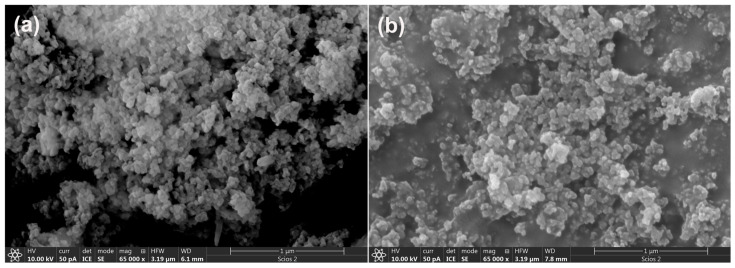
SEM micrographs of A_6,12_MKWA: (**a**) A_6_MKWA, and (**b**) A_12_MKWA.

Structural and phase analysis of the two-component MK-WA system indicates the formation of a predominantly amorphous aluminosilicate gel, coexisting with residual crystalline phases such as quartz, albite, calcite and kaolinite [23]. Compared to the pure precursors, the mixed system combines the high CaO content of WA with the Al_2_O_3_ and SiO_2_ from MK, promoting a stable gel network that integrates the mineralogical characteristics of both components [71]. In the two-component system of alkali-activated metakaolin (MK) and wood ash (WA), the aluminosilicate matrix contains non-uniformly distributed particle clusters smaller than 500 nm (Figure 9a). Increasing the NaOH concentration refines these clusters into smaller agglomerates (Figure 9b), although no substantial morphological changes are observed with varying activator concentration alone.

The addition of 10 wt.% WA into MK (sample AMKWA) induces subtle modifications in the microstructure, leading to the coexistence of dense gel blocks and irregular aluminosilicate gel structures (~4 μm) composed of small, clustered particles (Figure 9b). These formations are interspersed with a notably porous interstructural space. The presence of WA, particularly its calcium oxide content, acts as a key factor in shaping these surface morphological features, highlighting that the precursor composition has a more pronounced effect on matrix morphology than the concentration of the alkali activator (Figure 9).

**Figure 10:**
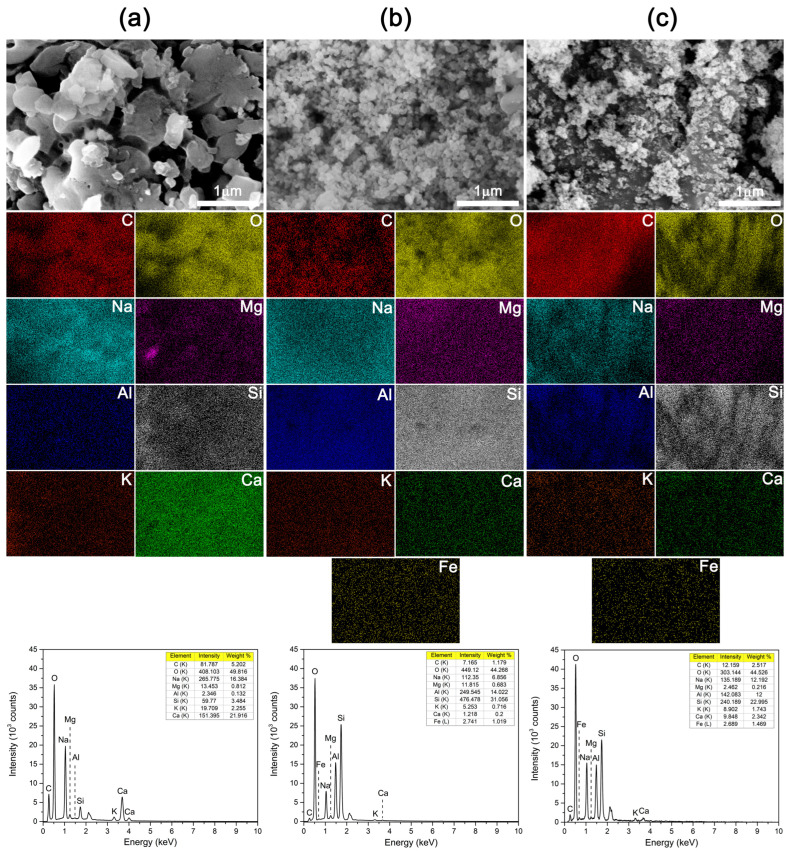
EDS elemental maps and corresponding spectra of: (**a**) A_12_WA, (**b**) A_12_MK, and (**c**) A_12_MKWA samples.

To further examine the elemental distribution across the surfaces of the investigated samples, EDS mapping was performed, and the corresponding maps and spectra for the A_12_WA, A_12_MK, and A_12_MKWA samples are presented in Figure 10.

For clarity, each element is represented by a specific color: carbon (C) in red, oxygen (O) in yellow, sodium (Na) in cyan, magnesium (Mg) in magenta, aluminum (Al) in blue, silicon (Si) in grey, potassium (K) in orange, and calcium (Ca) in green. In addition, iron (Fe) is depicted in light orange in Figure 10b,c. The obtained maps reveal a uniform spatial distribution of the detected elements across the examined areas of the material matrix, and the presence of all elements is further confirmed by the characteristic peaks in the corresponding EDS spectra. A variation in calcium content was observed among the samples, consistent with their respective compositions.

The A_12_WA sample (Figure 10a) exhibited the highest Ca content (21.916 wt.%), reflecting its intrinsic mineral composition, whereas the A_12_MK (Figure 10b) contained only trace amounts (0.2 wt.%). The A_12_MKWA sample (Figure 10c) showed a Ca content of 2.342 wt.%, corresponding to the proportional contribution of wood ash in the mixture. At high activator molarities, rapid dissolution and supersaturation promote partial precipitation of Ca-rich crystalline phases, limiting their incorporation into the continuous gel network. This disrupts gel continuity and may induce microcracking (see Figure 10c), reducing mechanical integrity. Calcium from wood ash, along with other reactive oxides, plays a key role in gel formation and matrix densification, but its dosage must be carefully controlled. Despite this, the compressive strength of AWAMK at higher pH of alkali activator remains only slightly lower than that of material activated with 6 M NaOH as part of the alkali activator, indicating that the microstructural benefits of higher alkalinity largely compensate for minor performance losses.

### 2.4. Radiological Analysis of AAMs

Radiological measurements were conducted on AAMs based on MK, WA, and MK–WA mixtures to evaluate health safety and their relevance for potential use in construction applications.

#### 2.4.1. Specific Activity of Radionuclides of AAMs

The specific activities of natural and artificial radionuclides in alkali-activated materials (AAMs) are presented here, providing insight into how the choice of precursor materials influences the radiological characteristics of the final products, as summarized in Table 5.

Precursor WA shows the specific activities for ^137^Cs (61.6 Bq/kg), while precursor MK shows this value is less than 0.1 Bq/kg (detectability limit) (Table 5). Cesium-137 (^137^Cs) is a man-made fission product with a physical half-life of ~30 years that originates from global fallout and other anthropogenic sources. As an artificial radionuclide, it can occasionally be detected at low levels in soils and recycled construction materials, but is not structurally or chemically active in the sense of altering the mechanical properties of binders or aggregates. Its presence in raw or secondary materials used for construction does not inherently compromise physical performance, though it can contribute to an external gamma dose field if present in significant concentrations. Regulatory guidance on radionuclides in building materials focuses on limiting the annual effective dose to the public to 1 mSv per year beyond outdoor background, regardless of radionuclide origin. This framework, adopted in International Atomic Energy Agency (IAEA) safety standards and related EU practice, is designed to ensure that materials, including those containing anthropogenic radionuclides, do not result in excessive radiological exposure when used in large bulk quantities, such as in roads or embankments. Assessment criteria (e.g., activity concentration indices) rely on measured activity concentrations (in Bq/kg) but apply the same dose limit irrespective of whether the radioactivity arises from natural or artificial sources. These criteria imply Cs-137 activity concentrations on the order of tens of Bq/kg, such as ~61 Bq/kg in the WA sample, are generally low and acceptable for typical civil engineering applications, provided the overall radiological assessment (including all radionuclides present) remains below the reference dose level.

A similar property was observed for the specific activity of ^40^K in the WA precursor (3840 Bq/kg) [71], which is a high value compared to the specific activity of ^40^K for the MK precursor, which was 641 Bq/kg [71]. Alkali activation of wood ash leads to a significant reduction in the activity of all radionuclides, especially with an increase in NaOH concentration in the alkali activator (Table 5). The activity of ^40^K before alkali activation was 3840 Bq/kg (Table 5) [71], while the activity values after activation are significantly lower, amounting to 1680 Bq/kg and 985 Bq/kg for A_6_WA and A_12_WA, respectively. It can be concluded that activation with 12 M NaOH in the alkali activator solution reduces the activity of ^40^K by approximately four times. Furthermore, a decrease in ^137^Cs activity is also noticeable, from 61.6 Bq/kg (Table 5) [71] to 22.1 Bq/kg and 18.4 Bq/kg (Table 5), with an increase in NaOH concentration in the alkali activator.

Table 5 shows the radionuclide activity values for alkali-activated metakaolin, where increasing the NaOH concentration in the alkali activator solution leads to a reduction in values. The activity of ^40^K decreases from 455 Bq/kg to 372 Bq/kg for A_6_MK and A_12_MK, respectively. M. Ivanović et al. (2018) used 16 M NaOH in the alkali activator for metakaolin activation and reported specific activity values of 154 Bq/kg for ^226^Ra, 55 Bq/kg for ^228^Ac, and 120 Bq/kg for ^40^K [72]. According to the results, it can be noted that the specific activity for ^228^Ac and ^40^K decreases with an increase in NaOH concentration in the alkali activator solution (Table 5). In the two-component system of metakaolin (MK) and wood ash (WA), the addition of 10 wt.% WA was noted to influence the presence of ^137^Cs, as this radionuclide was detected in WA in relation to MK. It is observed that increasing the NaOH concentration in the alkali activator solution increases the specific activity of ^137^Cs. In the case of specific activity of ^40^K, after the alkali activation process, the value of specific activity decreases. The lowest value of specific activity in a two-component system shows the A_6_MKWA for ^226^Ra.

#### 2.4.2. Radiological Hazard Indices of AAMs

To assess the radiological safety of the alkali-activated materials (AAMs), hazard indices were calculated based on the measured activity concentrations of radionuclides, providing a quantitative evaluation of potential exposure risks and ensuring compliance with recommended safety limits. Based on the specific activity values of natural radionuclides, corresponding hazard indices can be determined (see Section Assessment of Radiological Hazard). Table 6 presents the hazard indices of the precursors (WA and MK) [71] as well as those of the resulting AAMs, calculated from the respective radionuclide activities. Determining these values is important due to the potential use of these materials in the construction industry. Upon alkali activation of the precursors, a decrease in hazard index values is observed compared to the original precursors, and the influence of the NaOH concentration in the alkali activator solution on the hazard indices is also evident.

For the activation of WA, increasing the NaOH concentration of the alkali activator leads to a decrease in *I_γ_* and *H_ex_* values, which are less than 0.750 and 0.500 Bq/kg, respectively. During the alkali activation of raw materials, increasing the NaOH concentration in the activator solution leads to a decrease in *H_in_* values.

The annual effective dose rate (*EDR_in_*) for A_12_WA is 0.265 nSv/y. This represents a significant reduction compared to the raw WA (Table 5) [71], enabling the use of AAMs in construction. Alkali activation of WA reduces the absorbed dose rate (*Ḋ*), an effect most pronounced in the A_12_WA sample.

A significant decrease in *Ra_eq_* values was observed with increasing NaOH concentration in the alkali activator, up to A_12_MK, 238.0 Bq/kg, and the same trend was observed for other AAMs. The radiation hazard index (*H_ex_*) also decreased, to 0.643 Bq/kg for A_12_MK. Alkali activation of MK led to a decrease in the absorbed dose rate (*Ḋ*), which was most pronounced for A_12_MK. When M. Ivanović et al. (2018) activated metakaolin with 16 M NaOH, they obtained *Ra_eq_* and *H_ex_* values of 241.89 Bq/kg and 0.657, respectively [72]. Materials activated with 12 M NaOH yielded 238.0 Bq/kg and 0.643, indicating that further increases in NaOH concentration beyond 12 M do not significantly affect these indices.

After alkali activation of the raw two-component system of wood ash and metakaolin (Table 6), the activity concentration index (*I_γ_*) showed slightly lower values, but without a significant decrease with increasing NaOH concentration. A similar trend was observed for other hazard indices, with the notable exception of radium equivalent activity (*Ra_eq_*), which exhibited a significant decrease. According to our previous research [71], alkali-activated materials based on wood ash (90 wt.%, 80 wt.% and 70 wt.%) and metakaolin show *Ra_eq_* values below the recommended maximum level (370 Bq/kg), corresponding to an annual effective dose of less than 1 mSv.

Based on the activity concentration index (*I_γ_*), the potential applications of the material can be determined, i.e., whether it is suitable for indoor use or for outdoor civil engineering purposes. Alkali activation of the raw materials caused a decrease in *I_γ_* values, indicating that their use no longer poses a risk to public health. Furthermore, increasing the concentration of the alkali activator led to further decreases in *I_γ_* values.

The observed reduction in hazard indices after alkali activation of precursor materials can be attributed to the stabilization and immobilization of radionuclides within the geopolymer aluminosilicate matrix formed during activation. The geopolymerization process produces a three-dimensional network of interconnected Si–O and Al–O tetrahedra with negatively charged sites that can incorporate cationic radionuclides, including ^137^Cs, through ion-exchange and structural binding mechanisms, thereby reducing their mobility and leachability compared to the raw materials. Geopolymers have been widely investigated for radioactive waste immobilization, showing that radionuclides such as cesium can be effectively retained within the solid matrix with limited release upon leaching, indicating encapsulation within the amorphous and crystalline phases of the geopolymer network. The enhanced retention and reduced leachability of radionuclides explain the lower *Ra_eq_*, *H_ex_*, and activity concentration index values observed in alkali-activated materials compared to unreacted precursors [73,74].

IAEA review evidence indicates geopolymers’ capacity for radionuclide immobilization and enhanced performance vs. traditional cementitious materials [75,76].

## 3. Conclusions

This study comprehensively investigated the multifunctional properties of alkali-activated materials (AAMs) synthesized from different precursors, namely wood ash (WA) and metakaolin (MK), under varying concentrations of sodium hydroxide in the alkali activator solution. The results demonstrate that both the type of precursor and the concentration of the alkali activator critically influence the physicochemical, microstructural, mechanical, and radiological characteristics of the resulting materials. Structural and mineralogical analyses using DRIFT and XRD confirmed the formation of predominantly amorphous geopolymer networks accompanied by characteristic crystalline phases, with notable modifications in the gel structure correlating with increases in NaOH concentration. Mechanical properties, such as compressive strength, were significantly affected by precursor type and NaOH concentration, demonstrating improved reactivity and polymerization in MK-based systems at higher NaOH levels. In the case of WA, this is not necessarily true. Although, WA is generally considered as filler it may also contribute to the gel formation of additional phases depending on its chemical composition. The highest compressive strength was observed in MK-based AAMs activated with 12 M NaOH, whereas WA-based materials required only 6 M NaOH for activation but still exhibited lower compressive strength compared to MK. The addition of WA to the mixture (MKWA) significantly enhanced compressive strength in the 6 M system, while in the 12 M system the MKWA composite displayed almost half the strength of pure MK, indicating a complex interaction between precursor type, activator concentration, and phase formation. Radiological assessments confirmed that all AAMs met recommended safety standards, highlighting their potential as multifunctional materials for sustainable construction and environmentally friendly applications.

## 4. Materials and Methods

### 4.1. Alkali Activation Process

Alkali-activated materials (AAMs) were synthesized using solid aluminosilicate precursors (Table 7), namely wood ash (WA) and metakaolin (MK). The precursors were activated with alkali activator solutions composed of sodium hydroxide (NaOH) and sodium silicate (Na_2_SiO_3_). Alkali activator solutions were prepared using NaOH solutions of two different molar concentrations (6 M and 12 M). The volumetric ratio of NaOH to Na_2_SiO_3_ was maintained at 1:1.5 for all mixtures. Each activator solution was prepared prior to synthesis and stirred on a magnetic mixer for approximately two to three hours to ensure homogeneity. The solid precursors, wood ash (WA) and metakaolin (MK), were subjected to alkali activation individually as pure components (100%). Subsequently, a composite system comprising 90 wt.% MK and 10 wt.% WA was prepared and similarly alkali-activated, with a mass ratio of solid precursors to alkali activator solution of 1.0. To improve mixture consistency and workability, distilled water was added after rapid homogenization at a water-to-binder ratio (w/b) adjusted individually for each of the three systems, where *w* refers to the mass of water and *b* to the mass of the mixture of activator and precursors.

**Figure 11:**
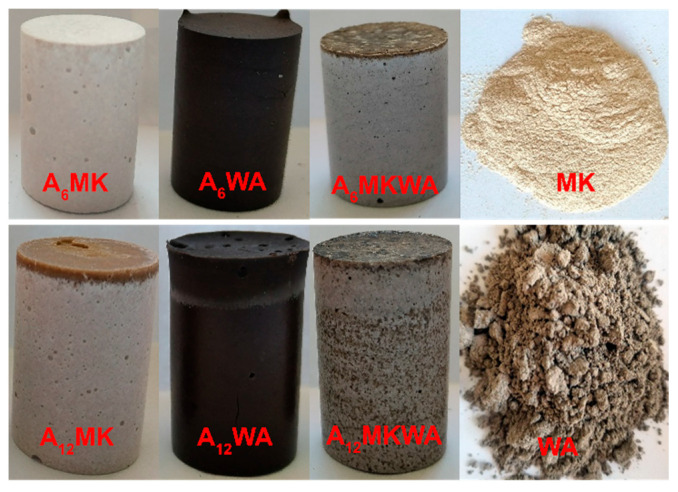
Photos of solid precursors and AAMs after 28 days of storage at room temperature.

The precursor materials and activator solutions were thoroughly mixed, poured into molds, and allowed to set for 24 h at room temperature (23 ± 1 °C). Subsequently, the samples were cured in an oven at 60 °C for 60 h, followed by storage at room temperature for a minimum of 28 days (Figure 11). After the curing period, the alkali-activated materials were crushed and sieved for subsequent analyses.

### 4.2. Method of Characterizations

The chemical composition of the investigated materials was determined by X-ray fluorescence (XRF) analysis. The measurements were conducted using a wavelength-dispersive X-ray fluorescence (WDXRF) spectrometer, ARL Perform’X (Thermo Scientific, Waltham, MA, USA), operating at 2500 W. The instrument was equipped with a 5GN Rh X-ray tube, four crystals (AX03, PET, LiF200, and LiF220), two detectors (proportional and scintillation), and the UniQuant software 5.lnk package for data processing. Sample preparation was carried out as described in our previous work [23].

The particle size distribution was measured using the Mastersizer 2000 instrument, Malvern Instruments Ltd., Malvern, UK, which works on the principle of diffracted light analysis [77].

Diffuse reflectance infrared Fourier transform (DRIFT) spectroscopy was employed to identify the functional groups present in the AAMs. The DRIFT spectra were recorded using a Spectrum Two FTIR spectrometer (PerkinElmer, Beaconsfield, UK). Spectra of the investigated samples were collected in the mid-infrared region (4000–400 cm^−1^) with a resolution of 4 cm^−1^.

The mineralogical composition of the AAMs was determined using X-ray diffraction (XRD) analysis. Measurements were performed on an Ultima IV diffractometer (Rigaku, Tokyo, Japan) over a 2θ range of 5–80°, with a step size of 0.02° and a scan rate of 5°/min in continuous scan mode. Phase identification and quantification were carried out using PDXL2 software version 2.8.4.0 (Rigaku, Tokyo, Japan) [53]. The obtained diffraction patterns were compared with reference data from the International Centre for Diffraction Data (ICDD) database, and the card numbers used to identify the phases were: kaolinite-PDF No: 01-072-5860, quartz-PDF No: 01-085-0865, calcite-PDF No: 01-071-3699, larnite-PDF No: 01-083-0464, nepheline-PDF No: 00-019-1176, faujasite-PDF No: 00-012-0246, muscovite-PDF No: 01-076-0928, mullite-PDF No: 00-015-0776, albite-PDF No: 00-009-0466, and illite-PDF No: 00-043-0685 [46].

Scanning electron microscopy (SEM) with an energy dispersive X-ray spectrometer (EDS) was employed to examine the morphology and elemental composition of the alkali-activated materials (AAMs). The analyses were conducted using the Scios2 DualBeam system (Thermo Fisher Scientific, Houston, TX, USA). Prior to imaging, the powder samples were mounted on aluminum stubs using double-sided conductive carbon tape and sputter-coated with a thin layer of gold to ensure surface conductivity. Micrographs and EDS elemental mapping were obtained at an acceleration voltage of 10 kV and a chamber pressure of approximately 9 × 10^−5^ Pa.

The compressive strength of the cylindrical AAMs was determined using an Instron M1185 universal testing machine at a loading rate of 1 mm/min. Prior to testing, the samples were carefully demolded and conditioned at room temperature (23 ± 1 °C) to ensure moisture equilibrium. The tests were conducted on specimens with a height-to-diameter ratio of approximately 2:1, following standard procedures for cementitious materials. Each measurement was performed in duplicate, and the average value was reported as the representative compressive strength for each sample.

### 4.3. Radiological Analysis

The assessment of natural radioactivity in building materials is crucial for evaluating their suitability for construction and ensuring radiological safety. Naturally occurring radionuclides from the uranium and thorium decay series and potassium-40 contribute to potential radiation exposure, necessitating the determination of their activity concentrations and related hazard indices (activity concentration index, radium equivalent activity, external and internal hazard indices).

Activity concentrations of ^226^Ra, ^232^Th, and ^40^K in AAMs were determined using semiconductor high-purity germanium (HPGe) detectors. Powdered samples were sealed in 125 mL PVC containers and stored for six weeks to reach radioactive equilibrium. Measurements were carried out with two coaxial HPGe spectrometers-AMETEK-ORTEC GEM 30–70 (37% relative efficiency, 1.8 keV resolution for ^60^Co at 1332.5 keV) and Canberra GX5019 (55% efficiency, 1.9 keV resolution). Energy and efficiency calibration were performed using a certified mixed gamma standard (CMI, Czech Republic) following IAEA recommendations [54,55]. Spectra were analysed with Canberra Genie 2000 software, with corrections for background, dead time, and coincidence summing; final calculations were performed in Mathematica 5.2 (Wolfram Research, Inc., Champaing, IL, USA).

#### Assessment of Radiological Hazard

The activity concentration index (*I_γ_*) is based on the ^226^Ra, ^232^Th and ^40^K activity concentrations. Radium equivalent activity (*Ra_eq_*), the index has been defined, to obtain the sum of activities for comparison of specific radioactivity of materials that contain different radionuclides ^226^Ra, ^232^Th and ^40^K. The external gamma radiation absorbed dose rate, *Ḋ* (nGy/h), in air 1 m above the ground due to radionuclides ^226^Ra, ^232^Th and ^40^K in measurement samples. The formulas used for the calculation of the assessment of radiological are presented in Table 8.

The value *I_γ_* ≤ 1 corresponds to an annual effective dose of gamma radiation of less than 1 mSv [78,79,80]. The value of *Ra_eq_*, must be less than one, i.e., to keep the radium equivalent activity and annual dose under the permissible limits of 370 Bq/kg and 1 mSv, respectively [80,81]. The value of the external and internal hazard index must be lower or at least equal to one, so that the hazard of radiation originating from radon and its decay products remains insignificant and negligible [82].

**Table 8:** Formulas of the assessment of radiological hazard.

Hazard Index	Formula	References
The activity concentration index	$I_{\gamma }=\frac{A_{Ra}}{300Bq/kg}+\frac{A_{Th}}{200Bq/kg}+\frac{A_{K}}{3000Bq/kg}\leq 1$	[78,79]
Radium equivalent activity	${Ra}_{eq}=A_{Ra}+1.43A_{Th}+0.077A_{K}$	[81]
External hazard index	$H_{ex}=\frac{A_{Ra}}{370Bq/kg}+\frac{A_{Th}}{259Bq/kg}+\frac{A_{K}}{4810Bq/kg}\leq 1$	[83,84]
Internal hazard index	$H_{in}=\frac{A_{Ra}}{185Bq/kg}+\frac{A_{Th}}{259Bq/kg}+\frac{A_{K}}{4810Bq/kg}\leq 1$	[82]
The external gamma radiation absorbed dose rate	$\overset{•}{D}=0.462A_{Ra}+0.604A_{Th}+0.0417A_{K}$	[84,85]
The annual outdoors effective dose rate	${EDR}_{out}=Ḋ\;\times 8760\times 0.2\times 0.7\times 10^{-6}$	[86]
The annual indoors effective dose rate	${EDR}_{in}=Ḋ\;\times 8760\times 0.8\times 0.7\times 10^{-6}$	[83,87]

Where *A*_Ra_, *A*_Th_ and *A*_K_ are the activity concentrations in Bq/kg of the corresponding radionuclides in the building materials.

## Figures and Tables

**Table 1:** Chemical composition of precursors WA and MK [23].

Oxide (wt.%)	Na_2_O	MgO	Al_2_O_3_	SiO_2_	P_2_O_5_	SO_3_	K_2_O	CaO	TiO_2_	MnO	Fe_2_O_3_	ZnO	As_2_O_3_	BaO	L.O.I. *
WA	0.51	4.25	4.06	4.07	1.92	1.18	11.16	38.76	0.11	1.43	0.72	0.19	0.14	0.26	31.06
MK	0.17	0.53	31.23	60.85	0.03	/	2.29	0.40	0.65	0.01	1.92	0.01	0.08	0.05	1.61

/—not detected; * Loss on ignition.

**Table 2:** Chemical composition of alkali-activated materials.

Oxide (wt.%)	Na_2_O	MgO	Al_2_O_3_	SiO_2_	P_2_O_5_	SO_3_	K_2_O	CaO	TiO_2_	MnO	Fe_2_O_3_	As_2_O_3_	BaO	L.O.I. *
A_6_WA	9.37	2.45	2.94	16.80	1.18	0.30	5.69	25.72	0.04	1.36	0.18	0.15	0.15	33.52
A_6_MK	8.21	0.40	22.61	52.01	0.04	/	1.53	0.29	0.44	0.02	1.34	0.13	0.04	12.83
A_6_MKWA	7.20	0.63	20.92	48.16	0.16	0.03	2.26	3.33	0.42	0.17	1.23	0.11	0.05	15.24

/—not detected; * Loss on ignition.

**Table 3:** Particle size parameters of precursors.

Precursors	d_0.1_	d_0.5_	d_0.9_
WA	0.955	5.073	70.781
MK	4.054	122.118	383.889

**Table 4:** Particle size parameters of AAMs.

AAMs	d_0.1_	d_0.5_	d_0.9_
A_6_WA	14.517	120.636	321.955
A_6_MK	21.395	178.884	382.181
A_6_MKWA	7.939	122.793	331.630

**Table 5:** Specific activities of radionuclides of precursors and AAMs.

AAMs	Specific Activities of Radionuclides, Bq/kg				
	^137^Cs	^210^Pb	^226^Ra	^228^Ac	^40^K
WA *	61.6 ± 3.2	53.5 ± 4.1	35.3 ± 3.6	34.0 ± 3.0	3840 ± 195
A_6_WA	22.1 ± 1.2	43.9 ± 3.4	23.5 ± 2.8	19.8 ± 1.5	1680 ± 80
A_12_WA	18.4 ± 1.0	18.2 ± 2.6	15.7 ± 1.8	9.3 ± 1.4	985 ± 50
MK *	<0.1	119.7 ± 10.3	209.6 ± 12.7	105.4 ± 5.6	641 ± 33
A_6_MK	<0.1	115.5 ± 5.7	149.3 ± 9.1	72.4 ± 3.9	455 ± 23
A_12_MK	<0.3	102.1 ± 5.5	120 ± 12	62.5 ± 3.9	372 ± 20
WAMK	2.88 ± 0.44	131.0 ± 14.6	130.7 ± 10.7	76.4 ± 4.6	660 ± 34
A_6_WAMK	3.69 ± 0.48	114.5 ± 6.3	85.7 ± 6.4	68.8 ± 4.6	627 ± 33
A_12_WAMK	3.77 ± 0.43	95.1 ± 5.4	126.8 ± 11.9	62.2 ± 4.0	550 ± 24

* According to our previous research [71].

**Table 6:** The activity concentration index (*I_γ_*), radium equivalent activity (*Ra_eq_*), external hazard index (*H_ex_*), internal hazard index (*H_in_*), External gamma radiation absorbed dose value (*Ḋ*), annual outdoors effective dose rate (*EDR_out_*) and annual outdoors effective dose rate (*EDR_in_*) of precursors and AAMs.

AAMs	*I_γ_*	*Ra_eq_* (Bq/kg)	*H_ex_* (Bq/kg)	*H_in_* (Bq/kg)	*Ḋ* (nGy/h)	*EDR_out_* (mSv/y)	*EDR_in_* (mSv/y)
WA *	1.568	379.6	1.025	1.120	197.0	0.242	0.966
A_6_WA	0.737	181.2	0.489	0.553	92.9	0.114	0.456
A_12_WA	0.427	104.8	0.283	0.326	53.9	0.066	0.265
MK *	1.439	409.7	1.107	1.673	187.2	0.230	0.918
A_6_MK	1.011	287.9	0.778	1.181	131.7	0.161	0.646
A_12_MK	0.837	238.0	0.643	0.967	108.7	0.133	0.533
WAMK	1.038	290.8	0.785	1.139	134.1	0.164	0.658
A_6_WAMK	0.839	232.4	0.628	0.859	107.3	0.132	0.526
A_12_WAMK	0.917	258.1	0.697	1.040	119.1	0.146	0.584

* According to our previous research [72].

**Table 7:** The solid precursors for synthesis of AAMs.

Solid Precursor (wt.%)	Concentration of NaOH, mol/dm^3^		AAMs
WA(100)	6	12	A_6,12_WA
MK(100)			A_6,12_MK
MK(90) WA(10)			A_6,12_MKWA

## Data Availability

The data presented in this study are available on request from the corresponding author.
